# Impact of Tracer Dose Reduction in [18 F]-Labelled Fluorodeoxyglucose-Positron Emission Tomography ([18 F]-FDG)-PET) on Texture Features and Histogram Indices: A Study in Homogeneous Tissues of Phantom and Patient

**DOI:** 10.3390/tomography9050143

**Published:** 2023-09-27

**Authors:** Jonas Vogel, Ferdinand Seith, Arne Estler, Konstantin Nikolaou, Holger Schmidt, Christian la Fougère, Thomas Küstner

**Affiliations:** 1 Nuclear Medicine and Clinical Molecular Imaging, Department of Radiology, University Hospital of Tuebingen, Otfried-Mueller-Strasse 14, 72076 Tuebingen, Germany; 2 Diagnostic and Interventional Radiology, Department of Radiology, University Hospital of Tuebingen, Hoppe-Seyler-Str. 3, 72076 Tuebingen, Germany; 3 German Cancer Consortium (DKTK), Partner Site Tuebingen, and German Cancer Research Center (DKFZ), 69120 Heidelberg, Germany; 4 Cluster of Excellence iFIT (EXC 2180): “Image-Guided and Functionally Instructed Tumor Therapies”, Eberhard Karls University, 72076 Tuebingen, Germany; 5 Medical Faculty, University of Tuebingen, Geschwister-Scholl-Platz, 72074 Tuebingen, Germany; 6 Medical Image and Data Analysis Lab (MIDAS.lab), Diagnostic and Interventional Radiology, University Hospital of Tuebingen, Hoppe-Seyler-Str. 3, 72076 Tuebingen, Germany

**Keywords:** PET, radiomics, texture features, dose reduction, FDG

## Abstract

Background: Histogram indices (HIs) and texture features (TFs) are considered to play an important role in future oncologic PET-imaging and it is unknown how these indices are affected by changes of tracer doses. A randomized undersampling of PET list mode data enables a simulation of tracer dose reduction. We performed a phantom study to compare HIs/TFs of simulated and measured tracer dose reductions and evaluated changes of HIs/TFs in the liver of patients with PETs from simulated reduced tracer doses. Overall, 42 HIs/TFs were evaluated in a NEMA phantom at measured and simulated doses (stepwise reduction of [18 F] from 100% to 25% of the measured dose). [18 F]-FDG-PET datasets of 15 patients were simulated from 3.0 down to 0.5 MBq/kgBW in intervals of 0.25 MBq/kgBW. HIs/TFs were calculated from two VOIs placed in physiological tissue of the right and left liver lobe and linear correlations and coefficients of variation analysis were performed. Results: All 42 TFs did not differ significantly in measured and simulated doses (*p* > 0.05). Also, 40 TFs showed the same behaviour over dose reduction regarding differences in the same group (measured or simulated), and for 26 TFs a linear behaviour over dose reduction for measured and simulated doses could be validated. Out of these, 13 TFs could be identified, which showed a linear change in TF value in both the NEMA phantom and patient data and therefore should maintain the same informative value when transferred in a dose reduction setting. Out of this Homogeneity 2, Entropy and Zone size non-uniformity are of special interest because they have been described as preferentially considerable for tumour heterogeneity characterization. Conclusions: We could show that there was no significant difference of measured and simulated HIs/TFs in the phantom study and most TFs reveal a linear behaviour over dose reduction, when tested in homogeneous tissue. This indicates that texture analysis in PET might be robust to dose modulations.

## 1. Background

[18 F]-labelled fluorodeoxyglucose ([18 F]-FDG)-positron emission tomography (PET) enables tomographic information about glucose metabolism in vivo. It is therefore considered to be a valuable tool, especially in staging and monitoring oncologic diseases. As invasive biopsies cannot be obtained from all tumours in advanced oncologic diseases and, moreover, might represent only small parts of heterogeneous tumours at a certain point of time [1], reliable quantitative PET imaging parameters beyond tumour size or tracer uptake values are desirable [2]. Histogram indices (HI) and textural features (TFs), respectively, represent first and higher-order statistics from the image [3]. So, these features represent information beyond visual representation that is contained inside medical images. Using this additional information could lead to improved diagnostic, prognostic and predictive models for medical research and daily patient care, which was widely evaluated in the last decade [4]. In [18 F]-FDG-PET, a voxel wise analysis of tumours or metastases is thought to represent the spatial distribution of metabolically active areas. Recently published studies suggest that the information gained might correlate to histopathology, metastatic status and therapy outcome [5,6,7,8]. On the other hand, it could be shown that individual TFs are influenced significantly by segmentation techniques, reconstruction parameters and different quantization techniques or highly correlate with each other, raising the question about their individual robustness [9,10,11].

Besides detector sensitivity and reconstruction parameters, also the amount of injected activity can influence the image quality in PET [12], and for oncologic [18 F]-FDG-PET examinations, heterogeneous dose recommendations exist, e.g., in the respective European and American societies [13,14]. A randomized undersampling of list-mode data enables a retrospective intra-individual simulation of tracer dose reductions [15,16,17]. Volume of metabolic active parts in a tumour is usually defined by an, e.g., 40% threshold of its highest standardized uptake value [18]. Previously studies could show that reduction of tracer doses can influence the metabolic tumour volume, which is of great importance for the calculation of TFs [11].

The aim of this study was to evaluate the influence of tracer dose reductions on HIs and TFs in [18 F]-FDG-PET in a standardized NEMA phantom (phantom according to standard of the National Electrical Manufacturers Association) as well as in the liver tissue of patients. The liver was chosen as a target organ due to its homogenic texture and sufficient size, allowing us to analyse both small and large volumes of interest (VOIs) with comparable texture and to avoid any further source of heterogeneity and influence on the textural features, so changes in these features represent only a function of changes in tracer doses.

## 2. Material and Methods

For feature extraction, an in-house developed feature extraction toolbox [19] was used that extends upon pyradiomics [20] for feature visualization and analysis. In order to validate the feature extraction tool, the tool was first used to verify the features calculated in the phantom and then used for calculating features from healthy liver tissue in the patient dataset.

We simplified the feature extraction task by targeting only TFs. The basic idea of TFs is to quantify intra-homogeneity in a defined VOI and inter-diversity between VOIs (of same or different images). The TFs can be grouped into 1st order (HI) capturing global features, 2nd order (e.g., grey-level co-occurrence) capturing local changes and higher orders (e.g., grey-level run-length) capturing regional discriminators. A complete list of the used TFs is given in Table 1. Images are all converted to SUV and after evaluating different bin sizes in a first step, due to the best specificity for final examination, a fixed quantification bin size of 1E-4 SUV was used for all extractions.

### 2.1. PET Acquisition

PET list-mode data of patients and phantoms were acquired. All examinations were performed in a fully integrated PET/MR scanner (Biograph mMR, Siemens Healthineers, Erlangen, Germany). PET data were stored in list-mode and the dose reductions were simulated by a retrospective randomized undersampling as carried out by Gatidis et al. [15]. PET image reconstruction parameters were as follows: 3D ordered-subset expectation maximization (3D-OSEM), 2 iterations, 21 subsets and a 4 mm Gaussian filter. Image reconstruction parameters were the same in all simulated and measured doses to prevent influences from reconstruction [9,21].

### 2.2. Phantom Study

To eliminate influences of individual patient characteristics and to compare the effect of simulated and measured dose reductions on HIs and TFs, we performed a phantom study using a standardized phantom according to the National Electric Manufacturers Association. The phantom body was filled with a background activity of 5.3 kBq/mL [18 F]-fluoridine solution in water. One sphere of 22 mm diameter was filled with an activity 4 times higher than the background activity. The 22 mm diameter was chosen as a representative lesion size in metastatic patients. Five consecutive PET measurements with an acquisition time of 20 min each were performed in intervals of 55 min (~0.5 half-life times of [18 F]), and the first measurement was performed directly after calibration and set as 100%. Following 20 min measurements can be considered equivalent to dose reductions to 71%, 50%, 35%, and 25%. Besides the real measurements, we also applied the randomized undersampling of list-mode data [15,16,17] to evaluate the accuracy between real measurements and numerical simulations, as well as to investigate the stability and generalizability of the proposed features. Attenuation correction for the PET data from the phantom scan was performed using an CT-based attenuation map of the phantom. A VOI with a diameter of 22 mm was placed in the sphere and copied to all measured and simulated PET images to evaluate TFs, details are shown in Table 2.

### 2.3. Patient Study

**Figure 1:**
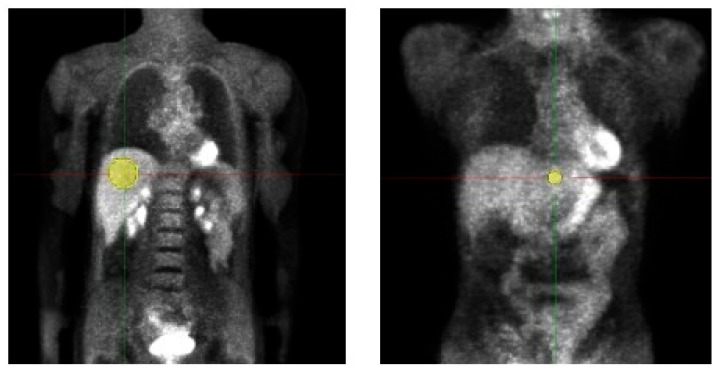
Volumes of interest (VOI) placement in patient study. Two VOIs were placed in healthy liver tissue of the left and right liver lobe and copied to all datasets of a patient for the analysis of HIs and TFs. VOI volume right liver lobe 106.8 ± 26.7 mL; left liver lobe 3.5 ± 0.9 mL.

We retrospectively evaluated the data of 19 adult patients (5 male/14 female, age 50.9 ± 11.7, Body weight (BW) 68.9 ± 11.9 kg) examined with a whole-body [18 F]-FDG-PET/MRI. Data acquisition was performed in the context of a prospective study (Ethic Committee: Ethik-Kommission an der Medizinischen Fakultät der Eberhard-Karl-Universität und am Universitätsklinikum Tübingen, Approval Code: 721/2012BO1, Approval Date: 14 March 2013) and all patients gave written informed consent concerning further clinical examination and scientific evaluation of their data. Before tracer injection, patients fasted for at least 6 h. The injected activity was at least 3 MBq/kgBW (as, e.g., also used in [10]) and the acquisition time per bed was 4 min. For attenuation correction, the vendor-provided Dixon-based segmentation method was used [22]. PETs were simulated with doses from 3 MBq/kgBW, which is the current diagnostic reference dose value for [18 F]-FDG-PET [23], down to 0.5 MBq/kgBW in intervals of 0.25 MBq/kgBW. Volumes of interest (VOIs) were placed in healthy liver tissue of the left and right liver lobe and copied to all datasets of a patient for the analysis of HIs and TFs, details are shown in Figure 1 and Table 2. Median VOI volume of VOI 1 (right liver lobe) was 106.8 ± 26.7 mL, respectively 3.5 ± 0.9 mL for VOI 2 (left liver lobe).

### 2.4. Statistical Analysis

Statistical analysis was performed using MATLAB (The MathWorks, Natick, MA, USA). For all tests, a *p*-value < 0.05 was considered as statistically significant. Correlation coefficient is grouped according to Cohen [24]: zero: abs(rho) < 0.1; weak: abs(rho) ≥ 0.1; medium: abs(rho) ≥ 0.3; strong: abs(rho) ≥ 0.5. All TFs possess a metric scale with a known zero point. Therefore, all statistical tests are based on ratio scales. In a first step, a Kolmogorov–Smirnov test was performed to evaluate the features for normal distribution.

#### 2.4.1. Phantom Study

Two dependent variables (measured and simulated doses) were compared, so if normally distributed, a paired sample *t*-test was performed to compare TFs from measured and simulated doses (H0 hypothesis: measured doses ≠ simulated doses). If normal distribution was not given, a Wilcoxon signed-rank test was performed.

Percentage dose deviations of reduced tracer doses (25–71%) for measured and simulated doses compared to the 100% dose value were calculated.

To test for a linear correlation between dose decay and changes in TFs, a Bravais–Pearson test (H0 hypothesis: not linearly correlated) was performed. If Bravais–Pearson test did not show a linear correlation Spearman test (H0 hypothesis: non-depended samples) was used to test on monotonic relationship. If no linear/monotonic correlation for original values could be shown additionally linear correlation for logarithmical values was tested.

#### 2.4.2. Patient Study

To test for the influence of dose modulation on TFs, linear mixed-effect models (LME) were used because requirements for a linear (regression) model were not fulfilled. To secure homoscedasticity and normal distribution of the models, residual analysis (plot of residuals vs. fitted values; normal probability plot of residuals) was performed and visually analysed. If homoscedasticity and normal distribution were not given, logarithmical TF values were tested. For each dose reduction step, a coefficient of variation (COV) of TF value change was calculated for each TF separately for VOI 1 and VOI 2 as

$$\mathrm{COV}=\frac{\mathrm{mean}\;\left({\mathrm{TF}}_{\left(3\mathrm{MBq}/\mathrm{kgBW}\right)}-{\;\mathrm{TF}}_{\left(\mathrm{reduced}\;\mathrm{tracer}\;\mathrm{dose}\right)}\right)}{\mathrm{standard}\;\mathrm{deviation}\;\left({\mathrm{TF}}_{\left(3\mathrm{MBq}/\mathrm{kgBW}\right)}-{\;\mathrm{TF}}_{\left(\mathrm{reduced}\;\mathrm{tracer}\;\mathrm{dose}\right)}\right)}$$

For evaluating the general variability of a single TF, these COVs were summarized for each TF over all dose reduction steps and both VOIs.

## 3. Results

### 3.1. Phantom Study

**Figure 2:**
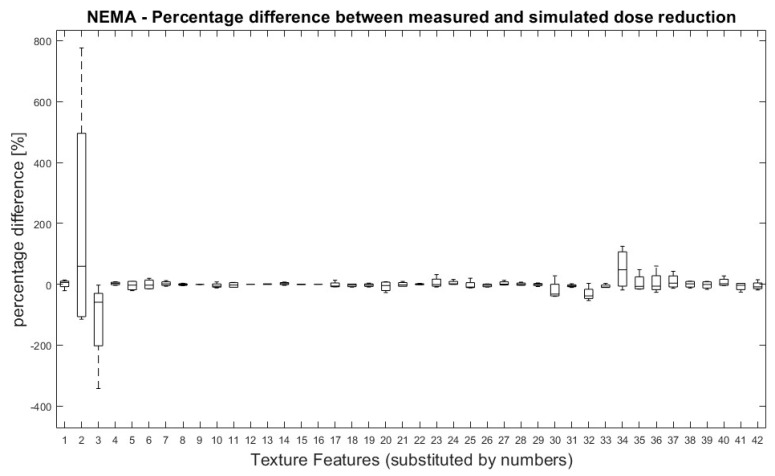
NEMA phantom: Percentage differences between measured and simulated doses (100%, 71%, 50%, 35% and 25%) for all dose pairs and texture features. Percentage differences between four measurements of measured and simulated doses for all TF. Measured dose values were used as references for percentage calculation. Allocation of TF and numbers can be performed by Table 1.

All TFs were normally distributed except for ‘Variance’ (first order), GLRLM LRHGE, GLZSM HGZE and GLZSM SZHGE. Overall, no significant differences were found between TF values derived from measured and simulated doses. Figure 2 shows the percentage difference between measured and simulated doses for all pairs of measurement (100% dose-reduced dose) and for all TFs.

**Figure 3:**
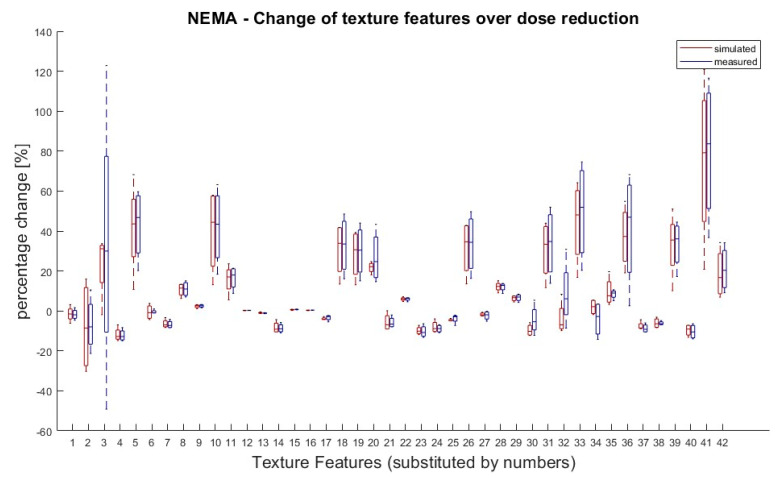
NEMA phantom: Percentage changes of texture features in simulated and measured doses for dose reductions (71%, 50%, 35% and 25%) in relation to 100% dose. TF value of reduced tracer doses (25–71%) for measured and simulated doses were compared to the 100% dose value. A 100% dose value was used as reference for percentage calculation. Values greater than ± 25% are considered as significant deviation. Eight TFs (Skewness, Kurtosis, GLCM Contrast, GLZSM LZLGE, GLZSM LZHGE, GLZSM GLV, NGTDM Strength and GLZSM LZLGE) showed significant changes over dose reduction between measured and simulated doses. Allocation of TF and numbers can be performed by Table 1.

It was also tested if there was a significant difference in TF values between doses within the same group (measured or simulated). According to van Helden et al. [11], a difference between the groups of 25% for a single value compared to the starting value (100% activity) was rated as significant. Eight TFs (Skewness, Kurtosis, GLCM Contrast, GLZSM LZLGE, GLZSM LZHGE, GLZSM GLV, NGTDM Strength and GLZSM LZLGE) showed significant differences in their dose correlation between measured and simulated doses. Except of these eight, all the other TFs showed the same behaviour for the measured and simulated doses. The results are shown in Figure 3.

In the next step it was evaluated if values of TFs reveal a linear correlation to dose reduction. This was the case in 26 out of 42 TFs. Twenty-six of them showed a linear behaviour in the original values and an additional two in the logarithmic TF values. The effect size according to Cohen was strong for all linear features. The results are shown in the first column (NEMA phantom) of Table 3. Further detailed analysis for the obtained Pearson’s *p*-values on the linearity tests can be examined in the Supplementary Figures S1 and S2.

### 3.2. Patient Study

Using the LME revealed that 13 TFs (ten original values, three logarithmical values) that were shown to correlate linearly with the amount of dose reduction in the phantom study were also linear in the patient data. The results of the LME and phantom study are summarized in Table 3. Further detailed analysis can be found in Supplementary Tables S1 and S2.

**Figure 4:**
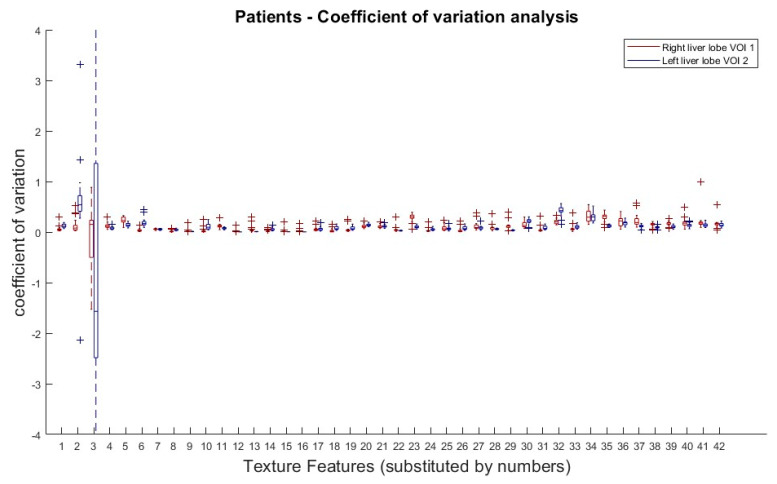
Patients: Coefficient of variation analysis over all dose reductions (simulated) in right (VOI 1) and left (VOI 2) liver lobe. Coefficients of variation (COV) of all dose reduction steps plotted for each TF and both VOIs. Allocation of TF and numbers can be performed by Table 1.

The results from the COV analysis are demonstrated in Figure 4. Obviously, there is no clear COV trend of any TF subgroup along the doses, with the exception of the first order TFs (Skewness and Kurtosis).

## 4. Discussion

The study intended to capture quantifiable changes in PET images in order to determine the optimal trade-off between sufficient and robust image quality and minimal required radioactivity. We hypothesized that a breakdown in image quality for too low counts, i.e., amplified noise (scatter and random incidents), might be reflected in textural changes of specified volumes of interest (VOI). We therefore focused on homogeneous tissue to exclude the potential bias caused by heterogeneity.

Using a NEMA phantom, it could be demonstrated that there are no significant differences between measured and simulated dose reduction using an undersampling algorithm as used in a previous study [15]. Most of the TF (34 of 42) showed the same behaviour in relation to dose reduction for measured and simulated data. Nevertheless, it has to be considered that in eight of these cases (GLCM Correlation, GLCM Entropy, GLRLM SRE, GLRLM LRE, GLRLM RLN, GLRLM RP, GLRLM LGRE and GLRLM LRLGE) differences could not have been detected due to the specific calculation of the TFs.

Test of linearity in relation to dose reduction showed different findings for six TF; however, *p*-values of measured and simulated data only differed slightly (<0.06) in four of the findings and should not be taken into account due to the small sample size. Only two TF showed a remarkable difference:

1. GLZSM LGZE (*p*-value-Δ = 0.21);
2. GLZSM ZSV (*p*-value-Δ = 0.117).

Overall, simulated dose reduction showed good accordance with measured data. It can hence be assumed that using the same undersampling algorithm for patient data should yield transferable findings to a real low dose PET examination.

For the patient data sets we could show that there is no evidence that there are certain TF metrics which are affected more or less by dose reduction. So, every TF has to be evaluated separately regarding its variation concerning (simulated) dose reduction.

Regarding the results of the NEMA phantom study and the patient datasets 13 TFs could be identified which showed a linear behaviour in both NEMA phantom and patient data and therefore could be considered as robust against tracer dose reduction, at least in homogenic tissue. They are shown in Table 4 and are sorted by summed COV. Because there are no guidelines for evaluating COVs of TF there is no clear cut-off, but only a trend for which TF are preferable in further studies. As mentioned, our phantom setup as well as the healthy liver both representing relatively homogenous tissue, therefore findings may not be seamlessly transferable to all kinds of tumour tissue. But as Gao et al. demonstrated in their study, TF analysis is feasible in dose reduction [18 F]-FDG-PET] and also in presumably inhomogeneous tumour tissue (primary lung cancer) [25].

There are several dozen studies investigating the clinical value of TF in a non-dose reduction setup [28,29]. Regarding these findings special interest should be taken on the following three TFs:

1. Homogeneity 2 (GLCM);
2. Entropy (GLCM);
3. Zone size non-uniformity (GLZSM).

We concur here with the findings of Tixier et al., who had proven a good physiological reproducibility and therefore classified them as parameters that should be preferably considered for tumour heterogeneity characterization [26]. Also, “Texture Strength” might be an interesting parameter due to Blanc-Durand et al. They generated a whole-liver radiomics scoring system based on [18 F]-FDG-PET for progression-free survival (PFS) and overall survival (OS) in patients undergoing trans-arterial radioembolization and therefore used “Texture Strength” as a sole parameter to predict OS and combined with three other parameters (Variance, GLCM Contrast, GLRLM SRLGE), which did not show a linear behaviour undergoing dose reduction, to predict PFS [27].

In this study, we could only address a small part of the potential interesting evaluations and so many aspects of this issue are not investigated yet. For example, we used a fixed bin size and did not evaluated the impact of bin size and lesion volume in a dose reduction setting, which had been shown to effect feature variations in non-dose reduction PET [30]. Also, we performed measurements with just one homogenies phantom and in homogenic liver tissue and did not include further phantoms, especially phantoms with textural phantom design or heterogenic (e.g., tumour) tissue to this study like it was performed by Gao et al. [25]. So, we have to acknowledge that our findings, which are only tested in healthy liver tissue, may vary for (heterogenic) tumour regions. These points, especially if the findings are transferable in tumour tissue, along with more basic tasks like test–retest reproducibility [26] or differences between different phantom types have to be evaluated further.

Due to the low number of studies that have investigated dose reduction in PET, generally all of the findings in this work could only be interpreted as first and provisional conclusions that have yet to be confirmed in further evaluations.

## 5. Conclusions

In this study, we showed that many TFs reveal a robust behaviour in homogenic tissue when undergoing dose reduction. We demonstrated that simulated dose reduction using a list-mode-based undersampling algorithm provides the same results as measured data in a phantom model when testing with 42 TFs.

Using simulated dose reduction in clinical indicated whole body [18 F]-FDG-PET, we also demonstrated that most of the tested TFs show a robust and linear behaviour over dose reduction in healthy liver tissue.

Therefore, TF analysis could also be used for low-dose PET, but this has to be further evaluated in future larger scale studies.

## Figures and Tables

**Table 1:** Extracted texture features.

Order	Scale	Metric	No.	Texture Feature
First	Global	Histogram Indices	1	Variance
			2	Skewness
			3	Kurtosis
Second	Local	Grey-level co-occurrence matrix (GLCM)/Grey-tone spatial dependency matrix (GTSDM)	4	Angular Second Moment/Energy
			5	Contrast
			6	Correlation
			7	Inverse difference moment/homogeneity 2
			8	Sum average
			9	Entropy
			10	Difference variance
			11	Dissimilarity
Higher	Regional	Grey-level run-length matrix (GLRLM)	12	Short run emphasis
			13	Long run emphasis
			14	Grey-level non-uniformity
			15	Run-length non-uniformity
			16	Run percentage
			17	Low grey-level run emphasis
			18	High grey-level run emphasis
			25	Short run low grey-level emphasis
			26	Short run high grey-level emphasis
			27	Long run low grey-level emphasis
			19	Long run high grey-level emphasis
			20	Grey-level variance
			21	Run-length variance
		Neighbourhood Grey-tone difference matrix (NGTDM)	38	Coarseness
			39	Contrast
			40	Busyness
			41	Complexity
			42	Texture Strength
		Grey-level Size Zone Matrix (GLZSM)	22	Small zone size emphasis
			23	Large zone size emphasis
			30	Low grey-level zone size emphasis
			31	High grey-level zone size emphasis
			32	Small zone/Low grey-level emphasis
			33	Small zone/High grey-level emphasis
			34	Large zone/Low grey-level emphasis
			35	Large zone/High grey-level emphasis
			24	Grey-level non-uniformity
			28	Zone size non-uniformity
			29	Zone size percentage
			36	Grey-level variance
			37	Zone-size variance

Overview of all 42 HI and TFs used in this evaluation. The number (No.) of the textural feature is determined by the used math lab algorithm and can be used to identify specific TFs in Figure 2, Figure 3 and Figure 4.

**Table 2:** Acquisition parameters.

	NEMA Phantom	Patient Study
Matrix size	344 × 344 × 128	256 × 256 × (418–709)
Pixel size [mm^2^]	2.8063 × 2.8063	2.8034 × 2.8034
Slice thickness [mm]	2.03125	2.0313
Quantification level	16 bit (65,536 grey levels)	
Mask size [voxel]	394	VOI 1 (right liver lobe): 5866 ± 1676 VOI 2 (left liver lobe): 223 ± 57

Acquisition parameter and mask size used for TF extractions in NEMA phantom and patient datasets.

**Table 3:** Linearity analysis.

		NEMA Phantom		Right Liver Lobe	Left Liver Lobe
No.	Feature	Measured	Simulated	(VOI 1)	(VOI 2)
**First Order (histogram indices)**					
1	Variance	-	-	-	-
2	Skewness	-	-	✔	✔
3	Kurtosis	-	-	-	-
**Second Order (GLCM)**					
4	GLCM Energy	✔	✔	-	-/✔ *
5	GLCM Contrast	✔	✔	-	-/✔ *
6	GLCM Correlation	-	-	-	-
7	GLCM Homogeneity 2	✔	✔	✔	✔
8	GLCM SumAverage	✔	✔	-	✔
9	GLCM Entropy	✔	✔	✔	✔
10	GLCM Variance	✔	✔	-	-
11	GLCM Dissimilarity	✔	✔	-	-/✔ *
**Higher order**					
12	GLRLM SRE	✔	✔	✔	✔
13	GLRLM LRE	✔	✔	✔	✔
14	GLRLM GLN	✔	✔	-	-/✔ *
15	GLRLM RLN	✔	✔	✔	✔
16	GLRLM RP	✔	✔	✔	✔
17	GLRLM LGRE	-	-	-	-
18	GLRLM HGRE	✔	✔	-	-
19	GLRLM LRHGE	✔ (p = 0.02)	- (p = 0.08)	✔	-
20	GLRLM GLV	✔	✔	-/✔ *	-
21	GLRLM RLV	✔ (p = 0.01)/✔	- (p = 0.06)/-	-/✔ *	-/✔ *
22	GLZSM SZE	✔	✔	✔	✔
23	GLZSM LZE	✔/✔	✔/✔	-/✔ *	✔/✔
24	GLZSM GLN	✔	✔	✔	✔
25	GLRLM SRLGE	-	-	✔	-
26	GLRLM SRHGE	✔	✔	-	✔
27	GLRLM LRLGE	-	-	-/✔ *	-
28	GLZSM ZSN	✔	✔	✔	✔
29	GLZSM ZP	✔	✔	✔	✔
30	GLZSM LGZE	- (p = 0.24)	✔ (p = 0.03)	-/✔ *	-
31	GLZSM HGZE	✔	✔	-	✔
32	GLZSM SZLGE	-	-	-/✔ *	-
33	GLZSM SZHGE	✔	✔	✔	✔
34	GLZSM LZLGE	-	-	-	-
35	GLZSM LZHGE	-	-	-/✔ *	-
36	GLZSM GLV	- (p = 0.05)/✔ *	✔ (p = 0.01)/✔	✔/✔	✔/✔
37	GLZSM ZSV	- (p = 0.12)	✔ (p < 0.01)	✔	✔
38	NGTDM Coarseness	✔/✔(p = 0.01) *	✔/- (p = 0.06) *	✔/✔	-/✔ *
39	NGTDM Contrast	✔	✔	-	-
40 41	NGTDM Busyness	✔	✔	-	-
41	NGTDM Complexity	✔	✔	-	-/✔ *
42	NGTDM Strength	✔ (p = 0.03)/✔	- (p = 0.09)/✔ *	✔/✔	✔/✔

*: No linearity regarding original but for logarithmical values (original/logarithmical). ✔: Linearity confirmed by Bravais-Pearson test. ✔: Monotonic correlation confirmed by Spearman test. -: No linear correlation. **☐** **☐**: Different findings for measured and simulated dose: only small differences regarding the number of measurements (black), high differences (red). Dark green: linear behaviour for NEMA and patient data. Light green: linear behaviour for NEMA data. For every TF linearity behaviour, over dose reduction was tested for measured and simulated NEMA phantom data as well as for patient data (big liver VOI and small liver VOI) with simulated dose reduction. Simulated and measured values in the NEMA phantom produce the same result in 36 of 42 TFs, with only minor differences in four cases and major differences in two cases. Overall, 26 TFs show linear behaviour in both measured and simulated phantom measurements, 13 of which also show linear behaviour in both liver VOIs.

**Table 4:** Texture features with linear behaviour over dose reduction in NEMA phantom and patient study.

No.	Texture Feature	Sum COV (VOI 1 + VOI 2)
7	Inverse difference moment/Homogeneity 2	44
28	Zone size non-uniformity	61
22	Small zone size emphasis	62
42	Texture Strength	73
15	Run-length non-uniformity	93
12	Short run emphasis	94
16	Run percentage	107
23	Large zone size emphasis	113
13	Long run emphasis	134
24	Grey-level non-uniformity	166
9	Entropy	173
36	Grey-level variance	238
29	Zone size percentage	820

Overview of all 13 TFs showing a linear behaviour over tracer dose reduction in NEMA phantom and patient study sorted by sum of coefficient of variation (COV) from all dose reduction steps and both VOIs. TFs with higher potential regarding usability in further studies are on top, but there is no existing cut-off value. Marked TFs (**dark green**) have been shown to have a good physiological reproducibility and therefore classified as preferentially considerable for tumour heterogeneity characterization by Tixier et al. [26]. TF marked (**light green**) was successfully used by Blanc-Durand et al. to predict outcome of patients undergoing [90 Y]-trans-arterial radioembolization (TARE) [27].

## Data Availability

All relevant data are within the paper and its Supporting Information files.

## Supplementary Materials

The following supporting information can be downloaded at: https://www.mdpi.com/article/10.3390/tomography9050143/s1, Figure S1: NEMA phantom: Linearity test on linear and logarithmic value scale for dose groups in simulated dose reductions of 71%, 50%, 35% and 25%. For every TF linearity behaviour over dose reduction was tested for measured and simulated NEMA phantom data. Pearson’s p-values for linear and logarithmic scale for simulated dose reduction are plotted. Figure S2: NEMA phantom: Linearity test on linear and logarithmic value scale for dose groups in measured dose reductions of 71%, 50%, 35% and 25%. For every TF linearity behaviour over dose reduction was tested for measured and simulated NEMA phantom data. Pearson’s p-values for linear and logarithmic scale for measured dose reduction are plotted. Tables S1 and S2: Patients: Linearity analysis for risht and left liver VOI: For every TF linearity behaviour over dose reduction was tested for patient data. Table S1 shows the results for the right liver VOI (volume 106.8 ± 26.7 mL), results for the left liver VOI (3.5 ± 0.9 mL) are displayed in Table S2. The results are also summarized in Table 3. Out of the 28 TFs which showed a linear behaviour in both measured and simulated NEMA phantom measurements 14 also showed linear behaviour in both liver VOIs. The estimated gradient was calculated for a step size of 0.25 MBq/kgBW.

## Abbreviations

[18 F]	Fluorine 18
BW	body weight
COV	coefficient of variation
CT	computer tomography
FDG	fluorodeoxyglucose
HI	histogram indices
MR(I)	magnetic resonance (imaging)
NEMA	National Electrical Manufacturers Association
OS	overall survival
OSEM	ordered subset expectation maximization
PET	positron emission tomography
PFS	progression free survival
TF	texture feature
VOI	volume of interest
