# Supplementary material for: Impact of Tracer Dose Reduction in [18 F]-Labelled Fluorodeoxyglucose-Positron Emission Tomography ([18 F]-FDG)-PET) on Texture Features and Histogram Indices: A Study in Homogeneous Tissues of Phantom and Patient
Source: Tomography. 2023 Sep 27;9(5):1799–810. doi: 10.3390/tomography9050143 (PMC10611106; doi:10.3390/tomography9050143)
Supplement: Supplementary file 1 [file tomography-09-00143-s001.zip › Suplementary Tabl. 1+2.pdf]

Table S1. Patients: Linearity analysis for right liver VOI

No.	Feature	Original values			Logarithmised values		
		linearity	p-value	estimated gradient	linearity	p-value	estimated gradient
**First Order (histogram indices)**							
1	Variance	-			-		
2	Skewness	✓	<0,001	-0,0777	-		
3	Kurtosis	-			-		
Second Order (GLCM)							
4	GLCM Energy	-			-		
5	GLCM Contrast	-			-		
6	GLCM Correlation	-			-		
7	GLCM Homogeneity 2	✓	<0,001	0,0253	-		
8	GLCM SumAverage	-			-		
9	GLCM Entropy	✓	<0,001	-0,2249	-		
10	GLCM Variance	-			-		
11	GLCM Dissimilarity	-			-		
Higher order							
12	GLRLM SRE	✓	<0,001	-0,0073	✓	<0,001	0,0077
13	GLRLM LRE	✓	<0,001	0,0638	-		
14	GLRLM GLN	-			-		
15	GLRLM RLN	✓	<0,001	-0,0162	-		
16	GLRLM RP	✓	<0,001	-0,0117	✓	<0,001	0,0129
17	GLRLM LGRE	-			-		
18	GLRLM HGRE	-			-		
19	GLRLM LRHGE	✓	<0,001	55,05	-		
20	GLRLM GLV	-			✓	<0,001	0,0780
21	GLRLM RLV	-			✓	<0,001	0,0728
22	GLZSM SZE	✓	<0,001	-0,0354	✓	<0,001	0,0557
23	GLZSM LZE	-			✓	<0,001	0,4252
24	GLZSM GLN	✓	<0,001	0,0004	✓	<0,001	-0,0215
25	GLRLM SRLGE	✓	0,011	-0,0005	✓	<0,001	0,0258
26	GLRLM SRHGE	-			-		
27	GLRLM LRLGE	-			✓	<0,001	-0,0725
28	GLZSM ZSN	✓	<0,001	-0,0407	✓	<0,001	0,1075
29	GLZSM ZP	✓	<0,001	-0,0586	✓	<0,001	0,1515
30	GLZSM LGZE	-			✓	<0,001	0,0874
31	GLZSM HGZE	-			-		
32	GLZSM SZLGE	-			✓	<0,001	0,1234
33	GLZSM SZHGE	✓	<0,001	-44,65	✓	<0,001	-0,0577
34	GLZSM LZLGE	-			-		
35	GLZSM LZHGE	-			✓	<0,001	0,4414
36	GLZSM GLV	✓	<0,001	-0,0004	✓	<0,001	0,1934
37	GLZSM ZSV	✓	<0,001	<0,0001	✓	<0,001	-0,1871
38	NGTDM Coarseness	✓	<0,001	0,0009	✓	<0,001	-0,2356
39	NGTDM Contrast	-			-		
40	NGTDM Busyness	-			-		
41	NGTDM Complexity	-			-		
42	NGTDM Strength	✓	<0,001	1,268	✓	<0,001	0,237

Table S2. Patients: Linearity analysis for left liver VOI

		Original values			Logarithmised values		
**No.**	**Feature**	**linearity**	**p-value**	**estimated gradient**	**linearity**	**p-value**	**estimated gradient**
**First Order (histogram indices)**							
1	Variance	-			-		
2	Skewness	✓	<0,001	-0,1296	-		
3	Kurtosis	-			-		
**Second Order (GLCM)**							
4	GLCM Energy	-			✓	<0,001	-0,0493
5	GLCM Contrast	-			✓	<0,001	-0-0938
6	GLCM	-			-		
7	GLCM	✓	<0,001	0,0044	✓	<0,001	-0,0310
8	GLCM	✓	<0,001	-0,0002	✓	<0,001	0,0252
9	GLCM Entropy	✓	<0,001	-0.0652	✓	<0,001	-0.0060
10	GLCM Variance	-			-		
11	GLCM	-			✓	<0,001	-0,0471
**Higher order**							
12	GLRLM SRE	✓	<0,001	-0,0009	✓	<0,001	0,0009
13	GLRLM LRE	✓	<0,001	-0,0036	✓	<0,001	0,0034
14	GLRLM GLN	-			✓	<0,001	-0,0345
15	GLRLM RLN	✓	<0,001	-0,0022	✓	<0,001	0,0023
16	GLRLM RP	✓	<0,001	-0,0011	✓	<0,001	0,0012
17	GLRLM LGRE	-			-		
18	GLRLM HGRE	-			-		
19	GLRLM LRHGE	-			-		
20	GLRLM GLV	-			-		
21	GLRLM RLV	-			✓	<0,001	-0,0421
22	GLZSM SZE	✓	<0,001	-0,0099	✓	<0,001	0,0117
23	GLZSM LZE	✓	<0,001	0,0771	✓	<0,001	0,0404
24	GLZSM GLN	✓	<0,001	0,0006	-		
25	GLRLM SRLGE	-			-		
26	GLRLM SRHGE	✓	<0,001	62,94	-		
27	GLRLM LRLGE	-			-		
28	GLZSM ZSN	✓	<0,001	-0,0170	✓	<0,001	0,0253
29	GLZSM ZP	✓	<0,001	-0,0121	✓	<0,001	0,0152
30	GLZSM LGZE	-			-		
31	GLZSM HGZE	✓	<0,001	-59,05	-		
32	GLZSM SZLGE	-			-		
33	GLZSM SZHGE	✓	<0,001	-59,40	✓	<0,001	-0,0569
34	GLZSM LZLGE	-			-		
35	GLZSM LZHGE	-			-		
36	GLZSM GLV	✓	0,01	-0,0027	✓	0,01	0,0477
37	GLZSM ZSV	✓	0,019	<0,0001	✓	0,03	-0,0255
38	NGTDM	-			✓	0,01	-0,0234
39	NGTDM Contrast	-			-		
40	NGTDM	-			-		
41	NGTDM	-			✓	<0,001	-0,1035
42	NGTDM Strength	✓	0,039	-0,9564	✓	0,03	-0,0273
