# Supplementary figures and images for: Impact of Tracer Dose Reduction in [18 F]-Labelled Fluorodeoxyglucose-Positron Emission Tomography ([18 F]-FDG)-PET) on Texture Features and Histogram Indices: A Study in Homogeneous Tissues of Phantom and Patient
Source: Tomography. 2023 Sep 27;9(5):1799–810. doi: 10.3390/tomography9050143 (PMC10611106; doi:10.3390/tomography9050143)

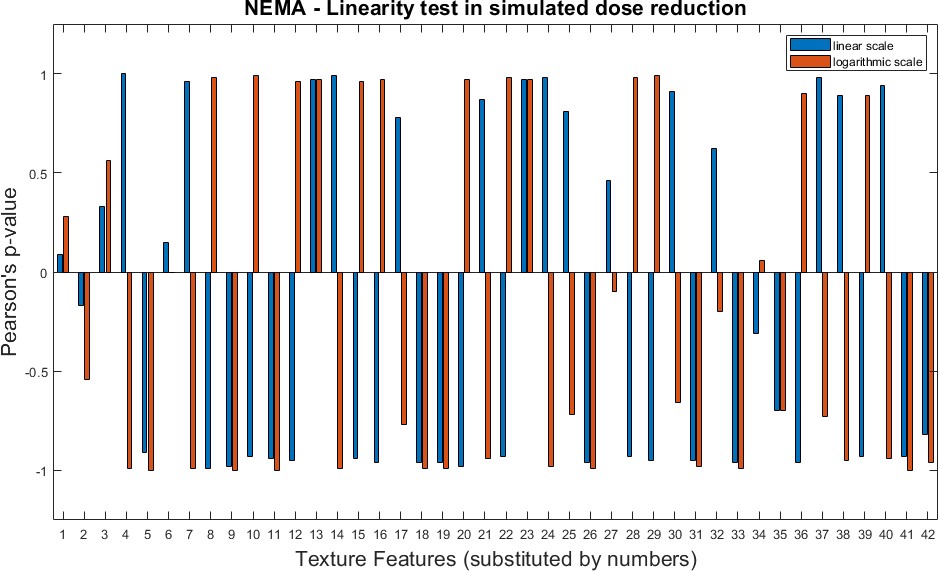

Supplement: Supplementary file 1 [file tomography-09-00143-s001.zip › Supl. Fig 1.jpg]

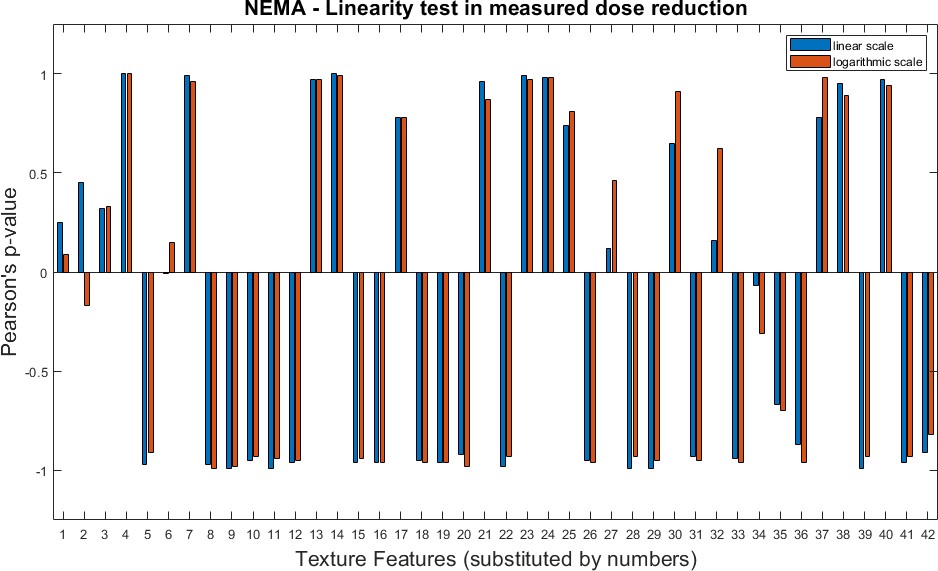

Supplement: Supplementary file 1 [file tomography-09-00143-s001.zip › Supl. Fig. 2.jpg]
